# Interspecies Anticancer and Antimicrobial Activities of Genus* Solanum* and Estimation of Rutin by Validated UPLC-PDA Method

**DOI:** 10.1155/2018/6040815

**Published:** 2018-07-03

**Authors:** Mohamed Fahad Alajmi, Perwez Alam, Md. Tabish Rehman, Fohad Mabood Husain, Azmat Ali Khan, Nasir Ali Siddiqui, Afzal Hussain, Mohd. Abul Kalam, Mohammad Khalid Parvez

**Affiliations:** ^1^Department of Pharmacognosy, College of Pharmacy, King Saud University, P.O. Box 2457, Riyadh 11451, Saudi Arabia; ^2^Department of Food Science and Nutrition, College of Food and Agriculture Sciences, King Saud University, Riyadh, Saudi Arabia; ^3^Department of Pharmaceutical Chemistry, College of Pharmacy, King Saud University, P.O. Box 2457, Riyadh, Saudi Arabia; ^4^Nanomedicine Research Unit, Department of Pharmaceutics, College of Pharmacy, King Saud University, Riyadh 11451, Saudi Arabia

## Abstract

Solanaceae is one of the highly diverse plant families of which* Solanum* is the largest genera (1700 species) containing several pharmacological properties like anticancer and antimicrobial. This motivated us to explore the anticancer (against HepG2, HEK-293, and MCF-7 cells) and antimicrobial (against* S. aureus, E. coli, P. aeruginosa, *and* C. albicans*) properties of* S. schimperianum*,* S. villosum*,* S. coagulans*,* S. glabratum*,* S. incanum,* and* S. nigrum* along with rutin estimation by UPLC-PDA method. Of the studied* Solanum* extracts,* S. nigrum *exhibited significant cytotoxic property against HepG2 (IC_50_: 20.4 *μ*g/mL) and MCF-7 (IC_50_: 30.1 *μ*g/mL);* S. coagulans* showed toxicity against HepG2 (IC_50_: 28.4 *μ*g/mL) and HEK-293 cells (IC_50_: 25.7 *μ*g/mL) compared to 5-Fluorouracil (standard). Compared to these, extracts of* S. coagulans* and* S. glabratum *exhibited relatively high antimicrobial potency (MIC: 0.4-1.6 mg/mL). Nonetheless, all* Solanum* extracts significantly reduced the biofilm against PAO1-strain. Rutin was detected in all extracts with the highest content (53.79 *μ*g/mg) in* S. coagulans* that supported its strong antimicrobial and anticancer properties. Molecular docking analysis showing strong binding of rutin with human DNA and proteins (DNA Topoisomerase II*α* and* E. coli* DNA gyrase B) supported the anticancer and antimicrobial activities of* Solanum* species.

## 1. Introduction

Solanaceae is one of the highly diverse plant families and consisted of wide range of therapeutically important chemical entities. It consists of perennial trees as well as herbaceous annual species, widely distributed from deserts to rainforests. Of this,* Solanum* is considered as the largest genera with nearly 1700 species. Sixteen species of* Solanum* are reported to be found in the West and Southwest areas of Saudi Arabia [1]. The extracts of* S. schimperianum* are reported to possess antioxidant, antitrypanosomal [2], and antimicrobial properties [3].* S. villosum* Miller extract showed strong in vitro cytotoxic property against breast cancer cells [4]. Also, proteins extracted from* S. villosum* leaves exhibited larvicidal properties against* Anopheles stephensi*,* Culex quinquefasciatus,* and* Stegomyia aegypti* and antimicrobial activities [5].* S. glabratum* Dunal extracts possessed excellent activity against intracellular amastigotes of* Leishmania infantum* [6] and strong cytotoxic property against breast (MCF-7), hepatocellular carcinoma (HepG2), and cervix (HeLa) cancer cells [7].* S. nigrum* Linn extract exhibited strong cytotoxic property against human malignant melanoma cell line (A-375) and breast cancer cells by [8].* S. nigrum* seed extracts exhibited moderate antiviral activity against hepatitis C virus NS3 protease [9]. Phytochemical investigation of* S. schimperianum* Hochst revealed the presence of a variety of steroidal, terpenoidal, and flavonoidal compounds like lupeol, *β*-sitosterol, *β*-sitosterol glucoside, oleanolic acid, ferutinin, 5-hydroxy-3,7,4'-trimethoxyflavone, retusin, and kaempferol-3-O-*β*-d-glucopyranoside [10] while* S. incanum* L. was reported to contain dioscin, protodioscin, methyl-protodioscin, indioside D, and solamargine. Incanumine, a steroidal alkaloid glycoside from* S. incanum,* has exhibited strong cytotoxic property against human PLC/PRF/5 cells in vitro [11].

**Figure 1:**
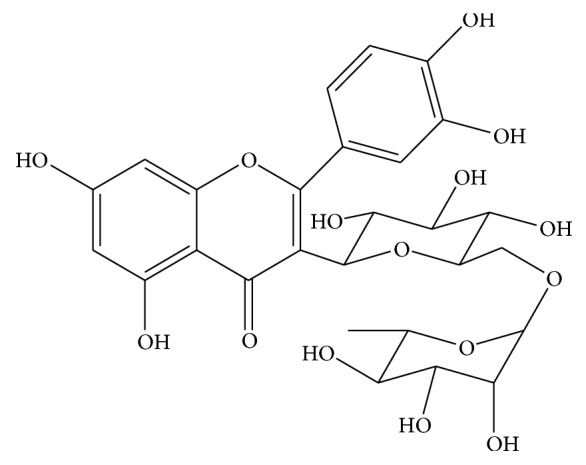
Chemical structure of rutin.

Since the isolated phytoconstituents and extracts of different species of* Solanum* were found to be very active against several microorganisms and cancer cell lines, it motivated the authors to investigate the antimicrobial and cytotoxic potential of aerial parts of six different species of genus* Solanum* grown in Saudi Arabia along with estimation of rutin (Figure 1) by validated UPLC-PDA method.

## 2. Materials and Methods

### 2.1. Plant Material

The aerial parts of* S. schimperianum* (Voucher no. 15308),* S. villosum* (Voucher no. 15032),* S. coagulans* (Voucher no. 15101),* S. glabratum* (Voucher no. 15043),* S. incanum* (Voucher no. 15102), and* S. nigrum* (Voucher no. 15149) were collected from Abha (Saudi Arabia) in 2014 and identified by the field taxonomist, Pharmacy College, King Saud University, Riyadh (KSA).

### 2.2. Chemicals and Reagents

The HPLC grade methanol and acetonitrile were purchased from Fisher Scientific, UK. Double-distilled, molecular grade water was obtained using a Millipore Milli-Q® (Bedford, MA, USA) water purifier. All the solvents (HPLC grade) and solutions were filtered through membrane filter (Millipore-Millex-HV® filter units, Durapore-PVDF®, polyethylene, 0.45 *μ*m pore size) and ultrasonicated for degassing before use. Other reagents used in the present study were of AR grade.

### 2.3. Instrumentation and Chromatographic Conditions

The ultra-performance liquid chromatography (UPLC) was performed on the gradient system of Agilent Technologies (1290 Infinity) equipped with G4220A Infinity Binary Pump and Autosampler, G1316C Thermostat Column Compartment and Fraction Collector with G4212A PDA (Photodiode Array) Detector. The programming of the above UPLC configuration was done by ChemStation software. The separation of analytes was carried out on Agilent ZORBAX Eclipse XDB 80Å C_18_ column (4.6 × 100 mm, 3.5 *μ*m; Agilent, California, USA) through gradient method by using acetonitrile and water as mobile phase. Other instruments used included vortex (VELP Scientifica, Italy), Mikro 200R centrifuge (Hettich Lab Technology North America), and Power Sonic 405 sonicator (Korea). All the analysis was performed at 25 ± 1°C temperature. The flow rate and injection volume were set at 0.18 mL/min and 10 *μ*L, respectively. The solutions and the mobile phases were sonicated for 30 min before use and UV-detection of rutin (analyte) was done at 332 nm.

### 2.4. Extraction of Plant Material by Ultrasonic-Assisted Method

The aerial parts of* S. schimperianum* (SS),* S. villosum* (SV),* S. coagulans* (SC),* S. glabratum* (SG),* S. incanum* (SI), and* S. nigrum* (SN) were air dried, powdered, and passed through a 0.75 mm sieve. The extraction process was accomplished in a Transsonic-460/H ultrasonic cleaner (ELMA, Germany). The powdered plant materials (20.0 g, each) were extracted for 30 min by ultrasonication (20 kHz, 240 W), using ethanol (95%) [12]. The obtained ethanol extracts of the six* Solanum* species (SSEE, SVEE, SCEE, SGEE, SIEE, and SNEE) were centrifuged at 5000 rpm for 20 min and filtered. All extracts were concentrated and dried under reduced pressure using rotary evaporator (R-210, BUCHI). The estimated yields (w/w) of SSEE, SVEE, SCEE, SGEE, SIEE, and SNEE were 9.71, 6.43, 5.92, 5.81, 3.75, and 8.52%, respectively.

### 2.5. In Vitro Anticancer Activity of the Ethanol Extracts of Different Solanum Species

#### 2.5.1. Cell Culture and Reagents

Human liver (HepG2), kidney (HEK-293), and breast (MCF-7) cancer cell lines were maintained in DMEM media supplemented with 10% bovine calf serum, 1X penicillin-streptomycin solution (Gibco, USA) at 37°C in a humidified chamber with 5% CO_2_ supply. 5-Fluorouracil (Sigma-Aldrich) was used as the reference anticancer drug. Dimethylsulfoxide (DMSO; Sigma-Aldrich, Germany) was used as vehicle.

#### 2.5.2. Cytotoxicity Assay

HepG2, HEK-293, and MCF-7 cells were seeded (0.5 x 10^5^ cells/well) in a 96-well flat-bottom plate (Becton-Dickinson Labware) and grown overnight. Each stock of SSEE, SVEE, SCEE, SGEE, SIEE, and SNEE was made by, first, dissolving in 100 *μ*L of DMSO and then in DMEM (1.0 mg/mL, final). Further, four different treatment doses (25, 50, 100, and 200 *μ*g/mL) were prepared in complete media. Next day, old media were discarded and cells were replenished with fresh media containing the extracts and 5-Fluorouracil (all in triplicate) and incubated for 48 h. Notably, the concentration of DMSO in the treatment doses never exceeded 0.1% and, therefore, was noncytotoxic. On day 2 after treatment, cytotoxicity test was performed, using TACS MTT Cell Proliferation and Viability Assay Kit (TACS) as per the manufacturer's instructions. Briefly, MTT reagent (10 *μ*L/well) was added to the cells and incubated for 3 h. The lysis buffer (100 *μ*L/well) was added and further incubated for 1.5-2 h. The absorbances (Abs; *λ*= 570 nm) were recorded in a microplate reader (BioTek, ELx800). The relationship between cell survival fraction and extract or drug concentration was plotted to obtain the survival curve of cancer cell lines, and the IC_50_ (*μ*g/mL) values were estimated using the best fit regression curve method in Excel (Microsoft, 2010).

#### 2.5.3. Microscopy

Any alterations in the morphology or growth of the treated cells were observed under an inverted microscope (Optika) at 24 and 48 h.

### 2.6. Antimicrobial Assay of the Ethanol Extracts of Different Solanum Species

#### 2.6.1. Antimicrobial Assay

The agar well diffusion method [13] as adopted earlier [14] was used. The diluted inoculum (0.1 mL; 10^5^ CFU/mL) of each test organism was spread on Muller-Hinton agar plates. 100 *μ*L (100 mg/mL) of each extract (SSEE, SVEE, SCEE, SGEE, SIEE, and SNEE) was loaded in wells of 8 mm diameter, DMSO as negative control separately. The plates were incubated overnight at 37°C followed by evaluation of antibacterial activity by measuring the zone of inhibition. Antibacterial agents (ampicillin and doxycycline) were used as positive controls while antifungal drug nystatin was used as the positive control for* Candida albicans*.

#### 2.6.2. Determination of Minimal Inhibitory Concentrations (MICs)

The minimal inhibitory concentrations (MICs) of the plant extracts were determined using standard method described by Eloff [15]. Briefly, the test samples were first dissolved in DMSO and the obtained solution was added to MHB and twofold dilution was performed (in a 96-well microtitre plate). Inoculum (100 *μ*L), i.e., approx. 1.5 × 10^6^ CFU/mL prepared in MHB, was then added to each well and incubated at 37°C for 18 h. Wells containing MHB, 100 *μ*L of inoculum, and 2.5% DMSO served as negative controls. The MICs of each extract were determined by adding 40 *μ*L p-Iodonitrotetrazolium chloride (0.2 mg/mL) and incubating at 37°C for 30 min. Reduction of yellow colored dye to pink indicated the presence of viable microbe. The MIC of each sample was defined as the lowest concentration that prevented this reduction.

#### 2.6.3. Assay for Biofilm Inhibition

The effect of test microbial agents on biofilm formation was measured using the microtitre plate assay [16]. Briefly, overnight grown cultures (OD = 0.4 at 600 nm) were added to 1 mL of fresh Luria Bertani broth (LB) medium in the presence or absence of sub-MICs of plant extracts and allowed to grow without agitation for 24 h at 30°C. After 24 h, media along with free-floating planktonic cells were emptied, and the wells were rinsed twice with sterile water. Adhered cells (biofilm) were stained with 200 *μ*L of 0.1% crystal violet (CV) solution (Hi-media, Mumbai, India). CV solution was discarded after 15 minutes and 200 *μ*L of 95% ethanol was added to solubilize CV from the stained cells. The absorbance was read at 470 nm in a microplate reader (Thermo Scientific Multiskan Ex, India) to quantify the biofilm biomass of biofilm.

### 2.7. Molecular Docking of Rutin with DNA

The three-dimensional coordinates of B-DNA dodecamer d(CGCGAATTCGCG)2 (PDB ID: 1BNA) were downloaded from RCSB Protein Data Bank [17]. The sdf files of rutin and 5-Fluorouracil (used as control) were downloaded from PubChem database (ID: 5280805 and 3385, respectively) and were converted into pdb format using OPENBABEL (http://www.vcclab.org/lab/babel). The water molecules and any heteroatoms were deleted from the receptor file (i.e., DNA) before setting up the docking program. The ligand and receptor files were energy-minimized using Charmm36 force field in Discovery Studio 4.0. The molecular docking of rutin and 5-Fluorouracil with DNA was performed using HEX 8.0.0 software with default settings except that the binding site of ligand on the receptor was searched on the basis of Shape as well as Electrostatics and the postprocessing was done by OPLS minimization. The GRID dimension was set at 0.6 and 10000 solutions were computed. The results were analyzed in Discovery Studio 4.0 [18].

### 2.8. Molecular Docking of Rutin with Proteins (DNA Topoisomerase II*α* and DNA Gyrase B)

MAESTRO (version 11.2, Schrödinger, LLC, New York, NY, USA) was used for all the steps involving protein and ligand preparation, receptor grid generation, and docking.

#### 2.8.1. Proteins Preparation

The X-ray crystal structures of kinase domains of human DNA Topoisomerase II*α* (PDB Id: 1ZXM, resolved 1.87 Å) and* E. coli* DNA gyrase B (PDB Id: 4KFG resolved at 1.60 Å) were downloaded from PDB database (http://www.rcsb.org/pdb). Protein preparation wizard of GLIDE was used for the assessment and refinement of protein structure before performing molecular docking. The structure of protein was prepared by removing water molecules, adding missing hydrogen atoms, assigning bond orders, creating zero bond order to disulfide bonds, and deleting any other heteroatoms expect respective ligands. Missing loops and any side chains were added using PRIME module of Schrödinger software. The protein was then optimized to create H-bond network and finally energy was minimized using the optimized potentials for liquid simulations 2005 (OPLS 2005) force field by setting a default constraint of 0.30 Å root mean square deviation (RMSD).

#### 2.8.2. Ligand Preparation

The structure of rutin was drawn using 2D sketcher of Schrödinger Suite and optimized for docking by assigning the bond orders and angles using LigPrep module. In LigPrep module, the 2D structure of rutin was converted into 3D structure and the energy was minimized using OPLS2005. The ionization state of rutin was generated at pH 7.0 ± 2.0 with the help of Epik module of LigPrep, keeping other parameters to default values.

#### 2.8.3. Grid Generation and Molecular Docking

The active site of human DNA Topoisomerase II *α* and* E. coli* DNA gyrase B was predicted using SiteMap module of Schrödinger software. The grid box was generated by selecting the centroid of the bound ligand as the centroid of the grid box. Molecular docking of rutin with DNA Topoisomerase and DNA gyrase B was performed with the help of GLIDE v.6.7 from the Schrödinger Suite. Standard precision (SP) and extra precision (XP) docking calculations were carried out and the parameters of scaling factor and partial charge cutoff were set at the default values 0.80 and 0.15, respectively. MM-GBSA (Molecular Mechanics-General Born Surface Area) calculation was performed for the estimation of binding energy. Postdocking analysis and visualization were performed on Maestro. The binding affinity of rutin with Topoisomerase and DNA gyrase B was calculated as described earlier [19, 20] using the following relation:

(1) $$\begin{array}{c} \Delta G=-RT\ln Kd \end{array}$$

where Δ*G* is the binding energy, T is temperature, R is Boltzmann gas constant (R=1.987 cal/mol/K), and* K*d is the binding affinity.

### 2.9. UPLC-PDA Analysis of Rutin in the Ethanol Extracts of Different Solanum Species

#### 2.9.1. Standard Solutions

The standard rutin (>94% pure) was procured from Sigma-Aldrich (USA). The accurately weighed quantity of rutin was dissolved in 5 mL of methanol in a 50 mL capacity volumetric flask and made up to 50 mL with methanol to get the stock (50 *μ*g/mL, final). Further dilution of the stock solution was done to get the final concentration of 1 *μ*g/mL.

#### 2.9.2. Sample Solutions

About 400 mg of the extract (SSEE, SVEE, SCEE, SGEE, SIEE, and SNEE) was dispersed in 10 mL of methanol and sonicated for its complete and quick dissolution. Subsequently, in a 10 mL capacity volumetric flask exactly 1250 *μ*L of the obtained solution was transferred and the volume was made up to 10 mL with the binary mobile phase to get a final known concentration of 5 mg/mL.

#### 2.9.3. Method Validation

The developed analytical method for the quantification of rutin in the SSEE, SVEE, SCEE, SGEE, SIEE, and SNEE was validated as per ICH guideline 2005 [21]. The validation parameters such as specificity, linearity, sensitivity, accuracy, precision, and recovery were checked. System suitability was tested in the beginning of quantitative analysis.

(*1) Specificity. *The ability of an analytical method to differentiate among the substances to be analyzed and other components present in the samples is known as specificity of the method. Specificity of the developed UPLC method is confirmed by elution and separation of the analytes of interest even in the presence of other potential constituents and the matrix. The resolution among the intensity of peaks of the desired constituents present in the SSEE, SVEE, SCEE, SGEE, SIEE, and SNEE was determined by analyzing the chromatograms obtained for reference standard and sample solutions, and the resolution was computed and estimated through the ChemStation software.

(*2) Linearity. *The linearity of the method was accessed by using the obtained calibration curves (*n* = 3) with the standard solutions at seven varying concentrations ranging from 1 to 80 *μ*g/mL for rutin. The values of the peak area obtained against the concentrations of the analytes were subjected to the linear regression analysis by using Excel (Microsoft, 2010).

(*3) Limit of Detection (LOD) and Limit of Quantification (LOQ). *The sensitivity of the developed method was established in terms of detection and quantification of the analytes, which was done from the calibration curves of the rutin. The LOD and LOQ were calculated by using the following equations:

(2) LOD=3.3SDS

(3) LOQ=10SDS

where SD was considered as the standard deviation of the responses and S was the slope of the calibration curve. LOD of any analytical method is the detection of the lowest amount of an analyte in any sample that can be noticed and identified but not essentially quantified, while LOQ of an analytical method is the quantification of the lowest amount of an analyte in any sample that can be calculated accurately with appropriate precision.

(*4) Interday and Intraday Precision. *The precision of the developed UPLC method was evaluated by assaying the samples. The assay of the samples was carried out in triplicate (*n* = 3) by adding known amounts of the standard solutions to the samples, at three different concentration levels (10, 20, and 40 *μ*g/mL for rutin) of the initial concentration of the sample. The intraday and interday precisions were articulated as the observed concentrations with respect to the actual concentrations in terms of relative standard deviations (RSD).

(*5) Recovery as Accuracy Study. *Recovery of the analyte by using the developed UPLC method was retrieved by analyzing the obtained peak areas of six determinations at four different concentration levels (20, 30, 40, and 50 *μ*g/mL for rutin). The variations in the recovered amount were stated in terms of percentage (%) of the obtained concentrations of the standards as well as in terms of relative standard deviations (%RSD).

(*6) Robustness. *Robustness of the method was determined by varying the wavelength parameter from 330 nm to 334 nm, by using columns from different suppliers and by changing the flow rate from 0.17 to 0.19 mL/min, for that three sample solutions were prepared and analyzed under the conditions established.

#### 2.9.4. Application of the Developed UPLC Method (Analysis of Rutin in Solanum Extracts)

The developed UPLC-PDA method was applied for the quantitative estimation of rutin in the alcoholic extract of six species of genus* Solanum *(SSEE, SVEE, SCEE, SGEE, SIEE, and SNEE). All the analytes were filtered through Milli-Q® filter unit before injecting in the UPLC system.

### 2.10. Statistical Analysis

The statistical analysis was performed by one-way analysis of variance (ANOVA) followed by Dunnett's test for the estimation of total variation in a set of data. Results were communicated in terms of mean values with ± SD where the probability (*p *< 0.01) of getting the result was considered as significant.

## 3. Results and Discussion

### 3.1. Anticancer Activity of Alcoholic Extracts of Different Solanum Species

The alcoholic extract of* S. schimperianum* (SSEE),* S. villosum* (SVEE),* S. coagulans* (SCEE),* S. glabratum *(SGEE),* S. incanum* (SIEE), and* S. nigrum* (SNEE) showed marked in vitro cytotoxic activities against human cancer cells (HepG2, HEK-293, and MCF-7) (Table 1). Of these, SNEE (IC_50_: 20.4 *μ*g/mL) and SCEE (IC_50_: 25.7 *μ*g/mL) had the most significant activity against human liver carcinoma cells (HepG2) while SIEE (IC_50_: 43.7 *μ*g/mL) possessed moderate activities. A saponin uttroside B isolated from* S. nigrum* leaves has been previously attributed for its excellent cytotoxic effect on HepG2 cells by downregulating the MAPK activation and mTOR pathways [22]. Solamargine and protodioscin (steroidal saponins) isolated from* S. coagulans* Forssk have exhibited strong in vitro cytotoxic property against liver and lung cancer cells [23, 24]. While SNEE showed excellent cytotoxic property against MCF-7 cells (IC_50_: 30.1 *μ*g/mL), SSEE, SCEE, and SGEE exhibited mild to moderate cytotoxicity. However, the rest of the* Solanum* extract was found ineffective. Moreover, SCEE exhibited strong cytotoxicity (IC_50_: 28.4 *μ*g/mL) against HEK-293 cells in comparison to the other tested extracts. Our findings therefore approve the strong cytotoxic property of SNEE (against HepG2, MCF-7, and MDA-MB-231 cells) and SCEE (against HepG2, HEK-293, and MCF-7 cells) compared to the other* Solanum* extracts.

### 3.2. Antimicrobial Assay of Alcoholic Extracts of Different Solanum Species

**Figure 2:**
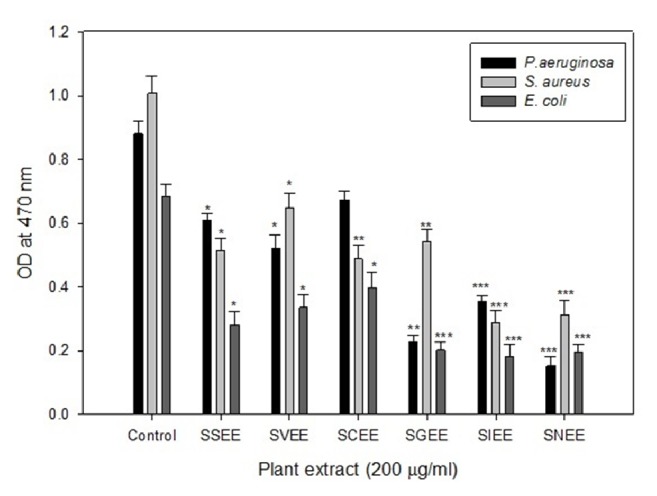
Effect of plant extracts on biofilm formation in pathogenic bacteria at subinhibitory concentrations. *∗*p ≤ 0.05; *∗∗* p ≤ 0.005; *∗∗∗* p ≤ 0.001.

All* Solanum* extracts (SSEE, SVEE, SCEE, SGEE, SIEE, and SNEE) demonstrated varying level of antibacterial and anticandidal activities in terms of zone of inhibition, ranging from 11 to 22 mm against bacterial strains* S. aureus*,* E. coli*, and* P. aeruginosa* and fungal strain* C. albicans* (Table 2). The MIC of SSEE, SVEE, SCEE, SGEE, SIEE, and SNEE ranged from 0.4 to 3.2 mg/mL (Table 3). Among the active extracts, SCEE, SGEE, SNEE, and SIEE showed relatively higher potency (MIC values from 0.4 to 3.2 mg/mL) against all the tested microbes. Because the MIC of crude extracts of individual plants varies against different test strains, the relationship between zone of inhibition and MIC may or may not be necessarily related. Since the crude extracts have mixture of phytoconstituents, they may influence the diffusion power of the active constituents. Several such observations are reported on using extracts of 15 higher plants used in Indian traditional medicine [25]. These differences could also be due to the differences in the chemical composition of these extracts as the secondary metabolites of plants have many effects including antibacterial and anticandidal properties. All* Solanum* extracts were further assayed for their biofilm inhibitory property against the* P. aeruginosa* PAO1 strain that demonstrated statistically significant reduction in biofilm as compared to the untreated control (Figure 2). Similar reduction in biofilm of PAO1 has previously been described with the extracts of edible plants and fruits of* Capparis spinosa* and* T. foenumgraceum* [26–28].

### 3.3. Molecular Docking of Rutin with DNA

**Figure 3:**
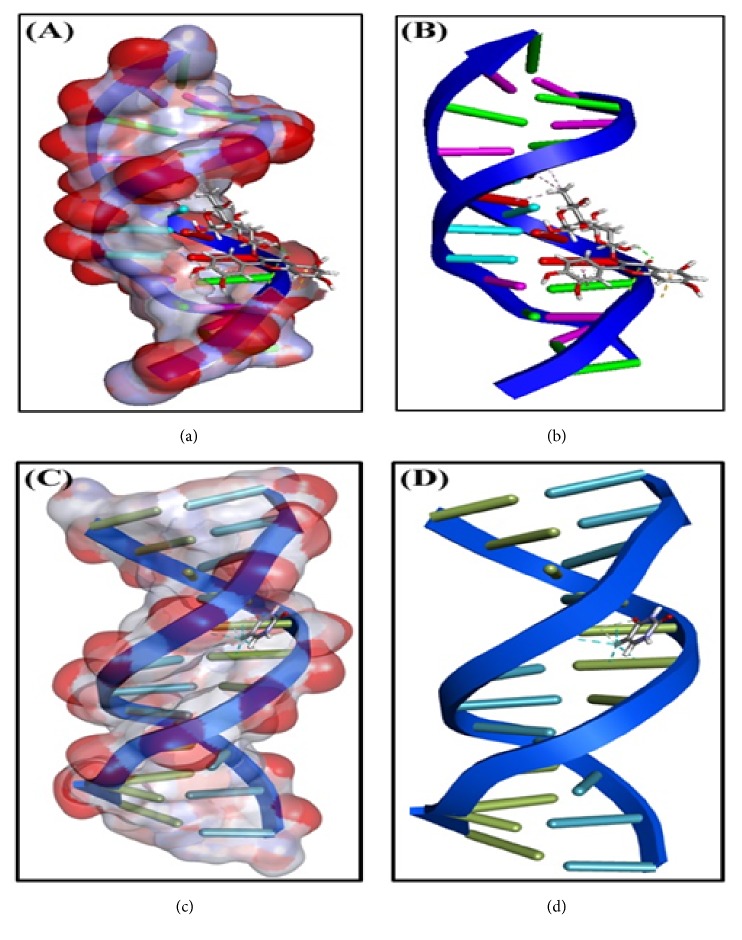
Interaction of DNA with rutin and 5-Fluorouracil (5-FU). Binding of rutin at major groove of DNA molecule ((a) and (b)) and binding of 5-FU at the minor groove of DNA ((c) and (d)).

In the present study, we have used* in silico* methods to probe the binding site of rutin on dodecameric DNA molecule and the molecular interactions involved in stabilizing the complex (Figure 3). The most preferred binding site of rutin on DNA was found to be located at the major groove of DNA with an overall binding score of -233.85. The complex was stabilized by one conventional hydrogen bonds OP2-atom with B: DA17 (2.30 Å), two carbon hydrogen bonds between A: DT7: O4 and H19 (2.36 Å) and H20 (2.35 Å) of rutin molecule (Table 4). Moreover, the DNA-rutin complex was stabilized by one electrostatic interaction between rutin and B: DG16: OP1 (2.66 Å) and one *π*-lone pair of interaction with B: DG16: OP2 (2.85 Å). Furthermore, C42 of rutin formed two hydrophobic (*π*-alkyl) interactions with A: DA5-atoms (3.68 Å and 5.31 Å) and one *π*-alkyl interaction with A: DA5-atom (4.15 Å) (Table 4). We also performed molecular docking of a control ligand, i.e., 5-Fluorouracil with DNA, and found that the overall binding score of 5-Fluorouracil with DNA was -144.63 (Table 4). It is therefore clear that rutin forms a strong interaction with DNA and could be used as an agent to block DNA replication.

### 3.4. Molecular Docking of Rutin with Human DNA Topoisomerase II*α*

**Figure 4:**
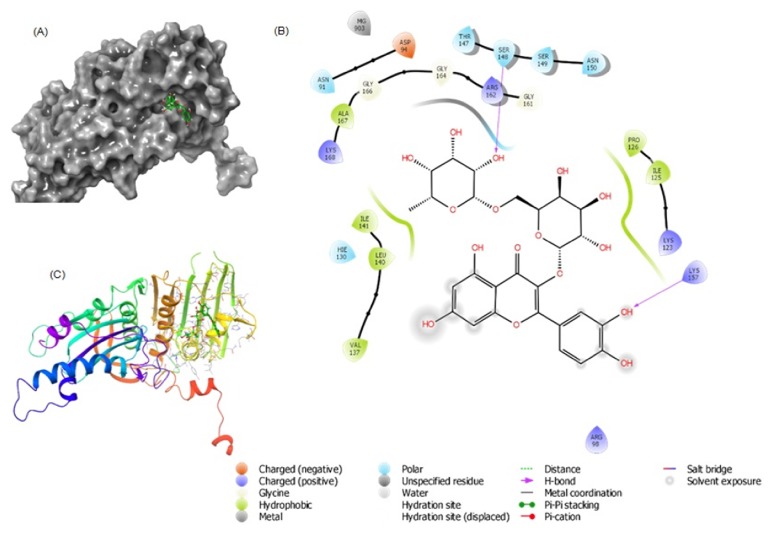
Molecular docking of rutin with human DNA Topoisomerase II*α*. (A) Binding of rutin at the ATP-binding site of Topoisomerase II*α*. (B) Molecular interaction of rutin with the residues of Topoisomerase II*α*. (C) Ribbon representation of the binding between rutin and Topoisomerase II*α*.

DNA Topoisomerase II is an essential enzyme that controls the topology of DNA during transcription, replication, recombination, and repair. It acts by cleaving and religating of both DNA strands [29]. Because of its pivotal role in cell viability, the Topoisomerase II*α* is an important target for several anticancer drugs [30]. The docking complex of rutin with Topoisomerase II*α* and the corresponding interacting amino acid residues are shown in Figure 4. It is evident that rutin formed a complex with Topoisomerase II*α* and the interactions were stabilized by two hydrogen bonds and six hydrophobic interactions. Rutin formed hydrogen bonds with Ser148 and Lys157, while residues such as Ile125, Pro126, Val137, Leu140, Ile141, and Ala167 formed hydrophobic interactions. Other residues involved in the interaction were Asn91, Asp94, Arg98, Lys123, Hie130, Thr147, Ser148, Ser149, Asn150, Gly161, Gly164, Gly166, and Lys168. It is interesting to note that rutin also interacted with Mg^2+^ which plays indispensable role in the catalytic activity of Topoisomerase II*α*. The docking score or binding energy of rutin-Topoisomerase II*α* interaction was estimated to be -6.901 kcal/mol in standard precision (SP) mode and -10.532 kcal/mol in extra precision (XP) mode which corresponded to the binding affinity of 1.15 × 10^5^/mol and 5.03 × 10^7^/mol, respectively (Table 5). Though the binding energy of rutin with Topoisomerase II*α* in XP mode was still lower than that of ANP-Topoisomerase II*α* complex (ΔG = -23.120 kcal/mol), rutin formed a high affinity and energetically favorable complex. Moreover, we also estimated the MM/PBSA (Molecular Mechanics energies combined with Poisson-Boltzmann or generalized Born and surface area continuum solvation) energies to accurately determine the strength of this interaction (ΔG = -96.481 kcal/mol). Our* in silico *data showing the strong rutin-DNA Topoisomerase II*α* interaction together with the high content of rutin in the extracts strongly supported the promising anticancer potential of* Solanum *species.

### 3.5. Molecular Docking of Rutin with Bacterial DNA Gyrase B

**Figure 5:**
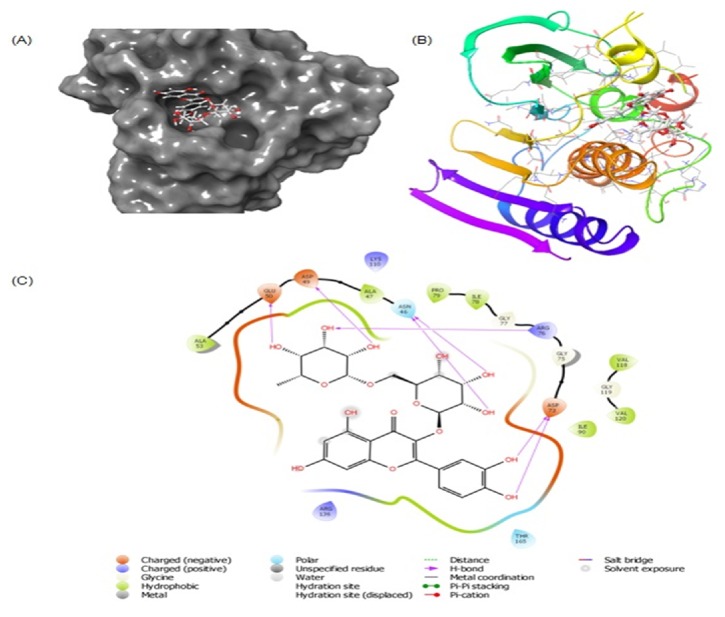
Molecular docking of rutin with bacterial DNA gyrase B. (A) Binding of rutin at the ATP-binding site of DNA gyrase B. (B) Molecular interaction of rutin with the residues of DNA gyrase B. (C) Ribbon representation of the binding between rutin and DNA gyrase B.

DNA gyrase and its homologs are present in prokaryotes and some unicellular eukaryotes, thus making it a good target for antibiotics. DNA gyrase has two subunits (A and B) that together bind to DNA, hydrolyze ATP, and introduce negative supertwists during replication. Two classes of natural anti-gyrase B aminocoumarins and quinolones are already reported [31] that work by competitive inhibition of energy transduction of DNA gyrase through ATPase activity. Docking of rutin with bacterial DNA gyrase B resulted in the generation of multiple poses of low energies, of which the best poses with lowest energies were further analyzed for the protein-ligand interactions. We compared the root mean square deviation (RMSD) between the published crystal pose and docked pose that was within the acceptable limit of 2.0 Å (Table 6). The docked complex of rutin with gyrase B and the corresponding interacting amino acid residues are shown in Figure 5. It was evident that rutin formed a complex with gyrase B and the interactions were stabilized by seven hydrogen bonds and seven hydrophobic interactions. Other residues involved in the interaction were Gly75, Gly77, Lys110, Gly119, Arg136, and Thr165. Rutin formed two hydrogen bonds each with Asp46 and Asp73, and one hydrogen bond each with Asp49, Glu50, and Arg76. The docking score or binding energy of gyrase B-rutin interaction was estimated in SP (ΔG = -7.065 kcal/mol) and XP (ΔG = -9.191 kcal/mol) modes which corresponded to the binding affinity of 2.13 × 10^6^/mol and 5.51 × 10^6^/mol, respectively. Moreover, we also estimated the MM/PBSA energies to accurately determine the strength of ligand-protein interaction (ΔG = -88.521 kcal/mol). In line with the* in silico *data on strong rutin-DNA gyrase B interaction, the high content of rutin in the extracts endorsed the marked antimicrobial activity of* Solanum *species.

### 3.6. UPLC-PDA Analysis of Rutin in Alcoholic Extracts of Different Solanum Species Optimization of Chromatographic Condition

**Figure 6:**
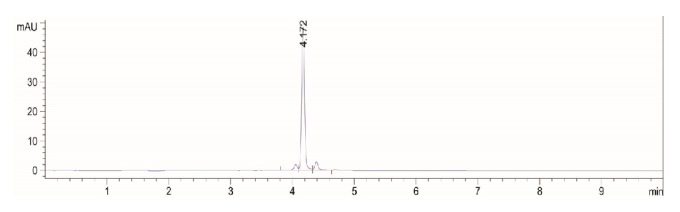
Representative chromatogram of rutin showing retention time at 4.172 min [Conditions: Eclipse XDB 80Å C18 column (4.6 × 100 mm, 3.5 *μ*m); mobile phase, acetonitrile: water (gradient system); flow rate, 0.18 mL/min; *λ*max = 332 nm at temperature (25 ± 1°C)].

Varying ratios of acetonitrile (Solvent-A) and water (Solvent-B) were used as mobile phase, column oven temperature, detection wavelength, and mobile phase flow rate were investigated for better separation of the analyte. A gradient program was employed for the separations of the active biomarker in one run within the reasonably short run time. The gradient was set up to achieve a linear increase of solvent-A from 0% to 10% for first 2 min followed by a steeper gradient, 10 to 35% of solvent-A in the next 3 min. Again the gradient was decreased from 35% to 20% in the next 2 minutes and finally, an additional 2 min of separation was extended with 100% solvent-B. The UV-detection wavelength of 332 nm was optimized and set as per the absorption maxima (*λ*_max_) for rutin. The developed method was found to furnish compact, sharp, and intense peak of rutin at the retention time (R_t_) = 4.172 min (Figure 6) with high resolution baseline.

#### 3.6.1. Method Validation

Calibration curves were linear over a large concentration range of 1-80 *μ*g/mL for rutin. Calibration curve exhibited good linear regression with coefficient of correlation (r^2^) value 0.9964 for rutin and recorded in Table 7. The LOD and LOQ obtained (Table 7) through the developed UPLC method were found as 0.015 and 0.047 *μ*g/mL, respectively, for rutin. The numerical values obtained for the intraday and interday precision were documented in Table 8. The developed method was found to be precise as the relative standard deviation (RSD) values for repeatability of intraday and interday precision studies were below 2.0%, which is under the limit as per recommendations of the International Conference on Harmonization guidelines. The recoveries in percentage and %RSD were deliberated from the numerical values of the y-intercept and slope of the obtained calibration curve. The calculated recovery values (Table 9) were found in the range of 98.67-99.20% for rutin, confirming the accuracy of the developed method. When the wavelength was intentionally changed from 330 to 334 nm, no any obvious effect was detected in the chromatogram, and no significant difference was found in the peak area and in retention time (Rt). However, there was a little-variation in retention time (Rt) obtained when the flow rate was varied from 0.16 to 0.20 mL/min. The low calculated values of standard deviations and %RSD with practically unaffected Rt values for rutin subsequently with small careful and deliberate changes as mentioned above indicated the robustness of the developed UPLC-PDA method (Table 10).

#### 3.6.2. Analysis of Rutin in SSEE, SVEE, SCEE, SGEE, SIEE, and SNEE

**Figure 7:**
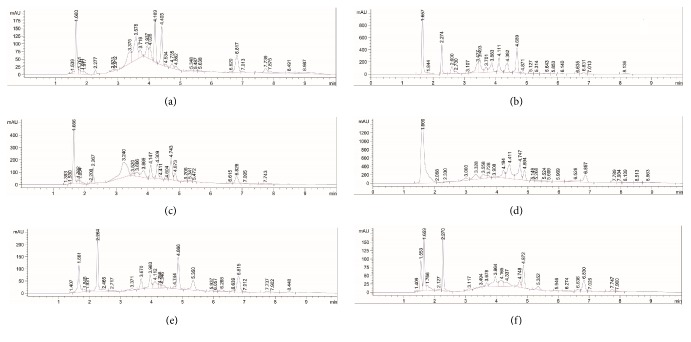
Representative chromatogram of rutin estimation in the ethanol extracts of* Solanum* species [Conditions: Eclipse XDB 80Å C18 column (4.6 × 100 mm, 3.5 *μ*m); mobile phase, acetonitrile: water (gradient system); flow rate, 0.18 mL/min; *λ*max = 332 nm at temperature (25 ± 1°C)]. (a) Representative chromatogram of SCEE showing rutin at Rt = 4.189 min. (b) Representative chromatogram SGEE showing rutin at Rt = 4.111 min. (c) Representative chromatogram of SIEE showing rutin at Rt = 4.147 min. (d) Representative chromatogram of SSEE showing rutin at Rt = 4.194 min. (e) Representative chromatogram of SNEE showing rutin at Rt = 4.112 min. (f) Representative chromatogram of SVEE showing rutin at Rt = 4.165 min.

The application of the developed UPLC method was evaluated by applying the method in the quantitative analysis of rutin in the ethanol extract of six species of genus* Solanum* (SSEE, SVEE, SCEE, SGEE, SIEE, and SNEE). A sharp, compact, and high resolution peak of rutin was observed in the chromatogram of SSEE, SVEE, SCEE, SGEE, SIEE, and SNEE samples. The quantity of rutin in SSEE, SVEE, SCEE, SGEE, SIEE, and SNEE was found as 4.995 ± 2.123, 0.433 ± 0.005, 53.797 ± 2.449, 21.991 ± 0.833, 19.501 ± 0.691, and 1.676 ± 0.017 *μ*g/mg of the dried weight of the extracts, respectively (Table 11). It was clearly evident from the result that, out of these six species,* S. coagulans* (Figure 7(a)) contained highest amount of rutin followed by* S. glabratum *(Figure 7(b)),* S. incanum* (Figure 7(c)),* S. schimperianum* (Figure 7(d)),* S. nigrum* (Figure 7(e)), and* S. villosum* (Figure 7(f)).

This might be considered as a maiden report on comparative analysis of rutin content in different* Solanum* species by using a precise, economical, and perfect UPLC-PDA method. Rutin has a high therapeutic value because it relieved the symptoms of several diseases like lymphatic and venous insufficiency, hemorrhagic diseases, and hypertension and due to its excellent hepatoprotective effect [32]. Though the cytotoxicity of rutin on cultured human cells is still debated, its marked cytotoxicity on different cancer cell lines has been widely reported [33–35]. It was found to induce apoptosis in prostate cancer cells, LNCaP at low concentration and in liver cancer cells, and HepG2 at very high concentration by acting as prooxidant instead of an antioxidant. It also inhibited the tumor cell growth by blocking cells amelioration through G0-G1 transition in HTC cells (rat hepatoma) by reducing the cells survival [36]. It is also reported to possess an excellent antibacterial property against* E. coli* by inhibiting the action of Topoisomerase II as well as its selective promotion of* E. coli* topoisomerase IV-dependent DNA cleavage, essential for viability [37]. In view of this, we have evaluated the anticancer and antibacterial properties of rutin only as it is one of the major components of Solanaceae family. In this report, high rutin content of* S. coagulans* supported its strong cytotoxic property against HepG2 and HEK-293. The moderate cytotoxic property of* S. incanum* and* S. schimperianum* may be attributed to its rutin content but the strong cytotoxic property of* S. nigrum* (SNEE) might be due to the synergistic effect of rutin along with other cytotoxic phytoconstituents like solamargine and protodioscin present in* S. nigrum*. However, elucidating the synergistic effect of other alkaloids with rutin is beyond the scope of this study. The highest zone of inhibition and low MIC value of SCEE, containing the highest amount of rutin, indicated the antimicrobial potential of rutin. In addition, our* in silico* molecular docking of rutin with DNA predicted the binding site and molecular interactions involved in the formation of a stable rutin-DNA complex.

## 4. Conclusion

In the present study, our results justified the screening of different species of genus* Solanum* traditionally used in folk medicine to treat several ailments like microbial infections and cancer. The aerial parts of* S. coagulans* and* S. nigrum* possessed significant cytotoxicity against breast and liver cancer cells compared to* S. schimperianum* and* S. glabratum*. On the other hand,* S. coagulans* extract was highly cytotoxic against kidney cancer cells compared to* S. schimperianum*. The extracts of all the* Solanum* species were found to possess marked antimicrobial activities against the tested strains. Further, the estimated high content of rutin in the* S. coagulans* supported its potent cytotoxic as well as antimicrobial properties. Since* Solanum* species contains numerous phytoconstituents which were proved to be highly cytotoxic and antimicrobial agents, our data warrants isolation of cytotoxic and antimicrobial compounds. Also, the developed UPLC-PDA method used to quantify rutin in* Solanum *species can be employed in the profiling of other genera and quality control of marketed herbal formulations.

## Figures and Tables

**Table 1:** The estimated IC_50_ (*μ*g/mL) values of ethanolic extracts of different species of genus *Solanum*.

Sample code	HepG2 (Liver)	HEK-293 (Kidney)	MCF-7 (Breast)
SSEE	58.3 ± 4.69	50.1 ± 4.18	42.1± 3.74
SVEE	69.4 ± 5.87	96.3 ± 6.98	71.1 ± 5.97
SCEE	25.7 ± 1.39	28.4 ± 1.41	45.2 ± 1.95
SGEE	58.2 ± 3.61	67.2 ± 3.79	49.3 ± 2.27
SIEE	43.7 ± 2.06	48.4 ± 2.11	65.3 ± 4.68
SNEE	20.4 ± 1.19	103 ± 7.98	30.1 ± 1.29
5-Fluorouracil	3.1 ± 0.09	2.5 ± 0.07	3.7 ± 0.10

*S. schimperianum* (SSEE),* S. villosum* (SVEE),* S. coagulans* (SCEE),* S. glabratum* (SGEE),* S. incanum* (SIEE),* and S. nigrum* (SNEE).

**Table 2:** Antimicrobial activities of ethanol extracts of different species of genus *Solanum*.

S. No.	Plant extracts	Zone of inhibition (mm)			
		*S. aureus*	*E. coli*	*P. aeruginosa*	*C. albicans*
1.	SSEE	19 ± 2.2	13 ± 0.9	16 ± 0.9	21 ± 1.4
2.	SVEE	22 ± 0.5	15 ± 2.5	21 ± 1.1	15 ± 1.2
3.	SCEE	20 ± 1.7	21 ± 0.8	17 ± 1.6	22 ± 0.9
4.	SGEE	14 ± 0.4	11 ± 0.3	15 ± 1	15 ± 0.6
5.	SIEE	18 ± 2.5	18 ± 1.6	12 ± 0.5	15 ± 0.8
6.	SNEE	11 ± 0.6	15 ± 0.2	16 ± 0.9	13 ± 0.4
7.	Ampicillin	21 ± 1.3	-	-	-
8.	Doxycycline	-	25 ± 1.6	24 ± 1.9	-
9.	Nystatin	-	-	-	23 ± 1.4

*S. schimperianum* (SSEE), *S. villosum* (SVEE), *S. coagulans* (SCEE), *S.glabratum* (SGEE), *S. incanum* (SIEE), and* S. nigrum* (SNEE).

**Table 3:** Minimum inhibitory concentration (MIC) of ethanol extracts of different species of genus *Solanum* against bacterial and fungal strains.

S. No.	Plant extracts	Minimum inhibitory concentration (mg/mL)			
		*S. aureus*	*E. coli*	*P. aeruginosa*	*C. albicans*
1.	SSEE	> 3.2	> 3.2	> 3.2	> 3.2
2.	SVEE	3.2	> 3.2	3.2	> 3.2
3.	SCEE	0.4	1.6	3.2	0.4
4.	SGEE	0.4	0.4	0.8	0.8
5.	SIEE	1.6	> 3.2	1.6	0.8
6.	SNEE	1.6	0.8	0.8	0.8

*S. schimperianum* (SSEE), *S. villosum* (SVEE), *S. coagulans* (SCEE), *S. glabratum *(SGEE), *S. incanum* (SIEE), and *S. nigrum* (SNEE).

**Table 4:** Molecular interactions between rutin and DNA.

Ligand atoms	DNA atom	Type of interactions	Bond distance (Å)	Docking Score
Rutin				

H12	B:DA17:OP2	Hydrogen Bond	2.30	-233.85
H19	A:DT7:O4	Carbon Hydrogen Bond	2.36	
H20	A:DT7:O4	Carbon Hydrogen Bond	2.35	
UNK1	B:DG16:OP1	Electrostatic (*π*-Anion)	2.66	
UNK1	B:DG16:OP2	*π*-Lone Pair	2.85	
C42	A:DA5	Hydrophobic (*π*-Alkyl)	3.68	
C42	A:DA5	Hydrophobic (*π*-Alkyl)	5.31	
C42	A:DA6	Hydrophobic (*π*-Alkyl)	4.15	

**5-Fluorouracil**				

F1	A:DA5:H2	Carbon Hydrogen Bond	2.33	-144.63
O2	A:DA5:H2	Carbon Hydrogen Bond	2.49	
F1	A:DA5:N3	Halogen bond	3.42	
F1	A:DA6:N9	Halogen bond	3.40	
F1	A:DA6:N3	Halogen bond	2.19	
F1	B:DT20:O2	Halogen bond	2.80	

**Table 5:** Interaction of rutin with Topoisomerase II*α*.

	Hydrogen bonds	Hydrophobic interactions	Other residues	Binding energy (kcal/mol)^#^	Binding affinity (M^−1^)	MM-GBSA (kcal/mol)
Topoisomerase II*α*	Ser148, Lys157	Ile125, Pro126, Val137, Leu140, Ile141, Ala167	Mg, Asn91, Asp94, Arg98, Lys123, Hie130, Thr147, Ser148, Ser149, Asn150, Gly161, Gly164, Gly166, Lys168,	-10.532	5.03 × 10^7^	-96.481

^#^binding energy in extra precision (XP) mode.

**Table 6:** Interaction of rutin with DNA gyrase B.

	Hydrogen bonds	Hydrophobic interactions	Other residues	Binding energy (kcal/mol)^#^	Binding affinity (M^−1^)	MM-GBSA (kcal/mol)
DNA gyrase B	Asn46*∗*, Asp49, Glu50, Asp73*∗*, Arg76	Ala47, Ala53, Ile78, Pro79, Ile90, Val118, Val120	Gly75, Gly77, Lys110, Gly119, Arg136, Thr165	-9.191	5.51 × 10^6^	-88.521

*∗*These residues form two hydrogen bonds; ^#^binding energy in extra precision (XP) mode.

**Table 7:** Retention time and linear regression data for the calibration curve and sensitivity parameters for rutin.

Parameters	For Rutin
Linearity range (*μ*g/mL)	1-80
Regression equation	*Y* = 2.2716*x* + 2.3916
Correlation coefficient (*r*^2^)	0.9964 ± 0.0004
Retention time (*R*_t_)	4.172 min
Slope ± SD	2.2716 ± 0.011
Intercept ± SD	2.3916 ± 0.046
LOD ( *μ*g/mL)	0.015
LOQ ( *μ*g/mL)	0.047

**Table 8:** Intra- and interday precision of the developed UPLC method for the analysis of rutin (mean ± SD, *n *=3).

Analyte	Nominal concentration (*μ*g/mL)	Intraday precision		Interday precision	
		Concentration detected (*μ*g/mL) ± SD	*∗*RSD (%)	Concentration detected (*μ*g/mL) ± SD	*∗*RSD (%)
Rutin	10.00	9.930 ± 0.061	0.620	9.798 ± 0.057	0.586
	20.00	20.029 ± 0.136	0.681	19.897 ±0.127	0.639
	40.00	39.446 ± 0.405	1.027	39.270 ± 0.398	1.014

*∗*RSD: relative standard deviation.

**Table 9:** Recovery of rutin for the accuracy of the developed method (mean ± SD, *n* = 3).

Percentage of rutin added (%)	Theoretical concentrations of rutin (*μ*g/mL)	Concentrations of rutin found (*μ*g/mL) ± SD	% RSD	% Recovery
0.0	20.0	19.841 ± 0.22	1.118	99.20
50.0	30.0	29.601 ± 0.35	1.188	98.67
100.0	40.0	39.446 ± 0.81	2.055	98.61
150.0	50.0	49.568 ± 1.10	2.235	99.13

**Table 10:** Robustness of the developed UPLC method (20 *μ*g/mL, of rutin, mean ± SD, *n* = 3).

Optimization conditions	Peak area for 20 *μ*g/mL of rutin		
	Mean ± SD	% RSD	Rt
Wavelength (*λ*_max_ = 332 nm)			

(330 nm)	50.253 ± 2.250	4.477	4.185
(332 nm)	49.972 ± 2.477	4.956	4.165
(334 nm)	49.649 ± 2.397	4.827	4.176

Mobile phase flow rate (0.18 ± 0.1 mL/min)			

(0.17 mL/min)	54.356 ± 2.528	4.650	4.181
(0.18 mL/min)	50.440 ± 2.366	4.690	4.172
(0.19 mL/min)	46.187 ± 2.269	4.912	4.167

**Table 11:** Detailed analysis report of rutin in the ethanol extract of different *Solanum* species.

Extract	Theoretical concentration of extracts (*μ*g/mL)	Concentration of rutin found in extract (*μ*g/mg) ± SD	% RSD	Rutin content (%)	Retention time (R_t_) (min)
SSEE	1000.0	4.995 ± 2.123	5.398	0.409	4.147
SVEE	1000.0	0.433 ± 0.005	1.154	0.043	4.112
SCEE	1000.0	53.797 ± 2.449	4.552	5.379	4.189
SGEE	1000.0	21.991 ± 0.833	3.787	2.199	4.111
SIEE	1000.0	19.501 ± 0.691	3.543	1.950	4.194
SNEE	1000.0	1.676 ± 0.017	1.014	0.167	4.112

*S. schimperianum* (SSEE), *S. villosum* (SVEE), *S. coagulans* (SCEE), *S. glabratum* (SGEE), *S. incanum* (SIEE), and *S. nigrum* (SNEE).

## Data Availability

All the required data are available in the manuscript.
